# Pulmonary lesion volume ratio and laboratory parameters as risk factors for plastic bronchitis in pediatric refractory *Mycoplasma pneumoniae* pneumonia

**DOI:** 10.1186/s12887-025-06381-2

**Published:** 2025-11-22

**Authors:** Jiahui Wu, Fangfang Cheng, Xiaoxing Kong, Qinghui Chen, Ting Shi, Yuanxi Bian, Jianmei Tian

**Affiliations:** Department of Infectious Diseases, Children’s Hospital of Soochow University, No. 92, Zhongnan Street, Suzhou, 215025 China

**Keywords:** Refractory *Mycoplasma pneumoniae* pneumonia, Plastic bronchitis, Risk factors, Pulmonary lesions volume

## Abstract

**Background:**

Refractory *Mycoplasma pneumoniae* pneumonia (RMPP) frequently leads to complications, including plastic bronchitis (PB). This study aimed to identify clinical risk factors for PB development in pediatric RMPP.

**Methods:**

A total of 361 pediatric patients with RMPP underwent bronchoscopy intervention were divided into a PB group and a non-PB group. Clinical characteristics, laboratory parameters, and chest CT findings were evaluated. Univariate analysis was initially performed to identify potential risk factors of PB, followed by multivariate logistic regression analysis to determine independent predictors, with receiver operating characteristic (ROC) analysis assessing their predictive value. A scoring system for PB risk assessment was developed based on odds ratio (OR) values.

**Results:**

PB patients showed significantly higher rates of extrapulmonary lesions (48.98% vs. 18.59%, *p* < 0.001), pleural effusion (59.18% vs. 25.64%, *p* < 0.001), and treatment resistance. Furthermore, PB patients demonstrated significantly elevated levels of neutrophil-to-lymphocyte ratio (NLR), C-reactive protein (CRP), procalcitonin (PCT), erythrocyte sedimentation rate (ESR), lactate dehydrogenase (LDH), aspartate aminotransferase (AST), alanine aminotransferase (ALT), D-dimer (DD), and pulmonary lesion volume ratio. Conversely, platelet count (PLT), prealbumin (PA), albumin (ALB), complement 3 (C3), complement 4 (C4), activated partial thromboplastin time (APTT), and the proportion of neutrophils in bronchial alveolar lavage fluid (BALF) were significantly lower compared to non-PB group (all *P*-values < 0.05). Multivariate analysis identified five independent predictors: NLR ≥ 3.30 (OR 2.92), LDH ≥ 451.0 U/L (OR 4.91), ALT ≥ 21.75 U/L (OR 5.68), pulmonary lesion volume ratio ≥ 8.23% (OR 8.83), and APTT ≤ 35.95s (OR 4.20). The combination of these predictors demonstrated strong diagnostic performance, with a sensitivity of 85.70%, specificity of 84.90%, and an area under the curve (AUC) of 0.92 for identifying PB cases. Using a scoring system based on ORs, we stratified 361 RMPP patients into risk groups: the high-risk group had 32 (60.38%) PB out of 53, the middle-risk group had 13 (22.02%) PB out of 59, and the low-risk group had 4 (1.61%) PB out of 249.

**Conclusions:**

Elevated NLR, increased LDH, higher ALT, greater pulmonary lesion volume ratio, and reduced APTT were identified as independent risk factors for PB. Pulmonary lesion volume ratio combined with key laboratory markers may facilitate early risk stratification and clinical intervention for PB in pediatric RMPP.

## Introduction

*Mycoplasma pneumoniae* pneumonia (MPP) is a common respiratory infection in children, caused by *Mycoplasma pneumoniae* (MP), a pathogenic bacterium lacking a cell wall [1]. MPP accounts for approximately 10–40% of community-acquired pneumonia cases in pediatric populations, with higher prevalence among children aged 5 years and above [2, 3]. While most cases resolve with macrolide antibiotics, a subset of patients develop refractory *Mycoplasma pneumoniae* pneumonia (RMPP), characterized by persistent fever, worsening clinical symptoms, and radiographic progression despite appropriate antimicrobial therapy for 7 days or more [4–6]. RMPP is associated with severe complications, including necrotizing pneumonia, pleural effusion, and plastic bronchitis (PB) [7].

PB is a rare but life-threatening complication of MPP, occurring when fibrin casts form in the bronchial tree, resulting in airway obstruction and respiratory failure. Studies have shown that PB can be detected and diagnosed in bronchial examinations for RMPP, with a reported incidence of 20–35% [7–9]. The presence of PB can result in atelectasis, wheezing, respiratory distress, and even death if casts are not removed in a timely manner [10, 11].

Several studies have demonstrated that persistent high fever, elevated lactate dehydrogenase (LDH) and Interleukin-6 (IL-6) levels, decreased platelet count (PLT), extrapulmonary complications, pleural effusion, and atelectasis serve as significant predictive indicators for PB formation in patients with RMPP [9, 12]. Radiological characteristics play a crucial role in evaluating RMPP occurrence, with consolidation and moderate-to-large pleural effusion being the most clinically relevant imaging features [13]. The most frequent chest CT findings in PB patients are atelectasis, followed by major airway obstruction [14]. Notably, current reported studies rarely employ pulmonary lesion volume ratio quantification based on chest CT for PB risk assessment in RMPP cases. Therefore, the aim of this study was to evaluate the value of pulmonary lesion volume ratio combined with laboratory parameters in identifying PB, which may guide early intervention strategies for RMPP patients.

## Materials and methods

### Study population

**Fig. 1:**
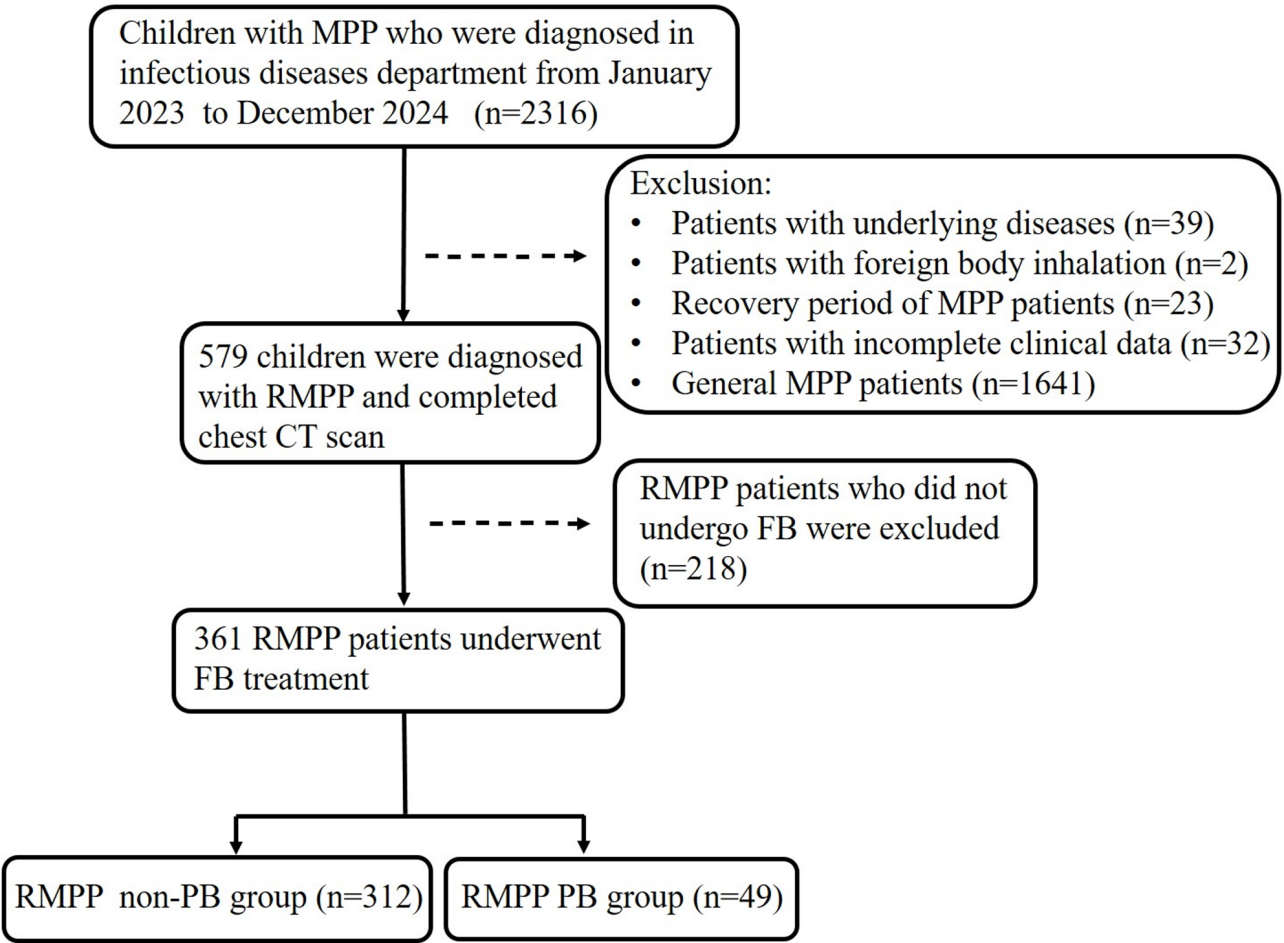
Flow chart of patients with refractory *Mycoplasma pneumoniae* pneumonia included in the study

This retrospective study was conducted at the Children’s Hospital of Soochow University and has been approved by our institutional ethics committee (Approval No. 2024-CS-119). The MPP patients hospitalized in the Department of Infectious Diseases between January 2023 and December 2024 met the diagnostic criteria of the MPP diagnosis and treatment expert consensus for children (2023 version) [15]. RMPP was defined as a condition in which pediatric patients with MPP exhibited persistent fever, unresolved or worsening clinical symptoms and radiographic findings despite receiving standard macrolide antibiotic therapy for over 7 days [4, 15]. For children diagnosed with RMPP who required fiberoptic bronchoscopy (FB) and bronchoalveolar lavage treatment, a complete chest CT scan was mandatory prior to the FB. Exclusion criteria: (1) patients with underlying diseases such as congenital heart disease, asthma, and immunodeficiency, (2) patients with a history of foreign body inhalation and confirmed by FB as tracheal foreign bodies, (3) patients readmitted due to other diseases during the recovery period of MPP, (4) patients with incomplete clinical data. The flowchart of our study was shown in Fig. 1.

### Data collection and definitions

All enrolled patients underwent related laboratory testing within 24 h of admission, including complete blood count, biochemical profile, humoral immunity, coagulation function, and nasopharyngeal aspirate pathogen screening. Bronchoalveolar lavage fluid (BALF) obtained through FB was routinely subjected to cytological classification. Clinical data were systematically collected, encompassing demographic information, bronchoscopy findings, therapeutic measures, and outcomes. All patients underwent the chest CT scan (GE Optima CT 660 scanner) in a supine position within 2 weeks of MP infection. CT images were acquired in axial, sagittal, and coronal planes with a slice thickness of 5 mm using lung window settings. The diagnosis of RMPP complicated with PB was based on bronchoscopic visualization of airway obstruction by inflammatory secretions, followed by removal of dendritic plastic casts using biopsy forceps or gentle suction. Histopathological examination confirmed that these casts consisted of inflammatory cells (mainly eosinophils and neutrophils) and shed epithelial cells. Extrapulmonary lesions of MPP were defined as involving systems beyond pulmonary lesions, mainly including gastrointestinal tract, nervous system, cardiovascular system, hematopoietic system, musculoskeletal system, skin and mucosal damage [7, 16]. Tetracyclines (doxycycline, minocycline) and fluoroquinolones (levofloxacin, moxifloxacin) served as alternative antibiotic treatment options for MPP [17]. Glucocorticoid resistant RMPP was defined as persistent or recrudescent fever >72 h after intravenous methylprednisolone of 2 mg/kg/day [18].

### Chest CT protocol

**Fig. 2:**
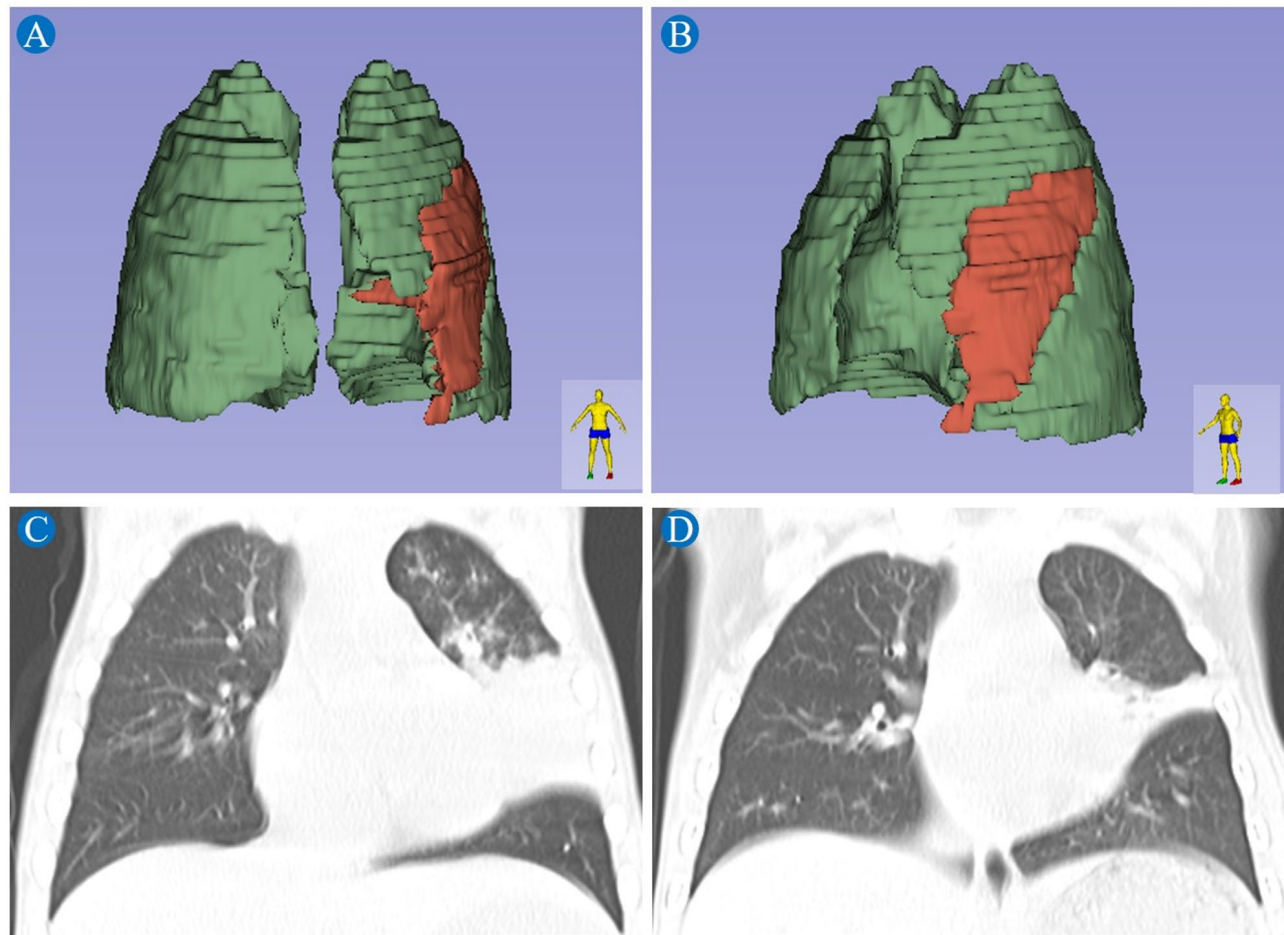
Images **A**-**D** depict a 7-year-old-boy with refractory *Mycoplasma pneumoniae *pneumonia complicated by plastic bronchitis. **A** Paramedian view of the lung reconstruction generated using 3D Slicer software based on manual segmentation. **B** Left lateral view of the affected lung, showing the distribution of diseased (red) and normal (green) tissues. The volume ratio of pulmonary lesions was calculated as the volume of diseased tissue divided by the total lung volume. **C** Pre-bronchoscopy chest CT (coronal view) showed extensive consolidation in the left upper lobe. **D** Follow-up chest CT one week post-bronchoscopy showed a patchy high-density shadow in the left upper lobe with marked radiographic improvement

Lung volume measurements were independently measured by 2 professional pediatric radiologists. The chest CT images of RMPP patients were imported into 3D Slicer software (version 5.2.1, www.slicer.org) in order to create lung profile and measure lung volume. The main steps were as follows: (1) imported the original CT images of RMPP patients in DICOM format, (2) ran the Editor module under the 2-dimensional window, (3) performed segmentations to trace the outer contours of the lung manually on each slide, (4) used the 3-D segmentation function of the program, the lung volume were calculated, (5) the volume of the red part divided by the sum of the volume of the red and green parts was the volume ratio of the pulmonary lesions (Fig. 2A-B).

### Statistical analysis

All statistical analyses were conducted using SPSS software (version 27.0). Normally distributed continuous data were presented as mean ± standard deviation and were compared between the two groups using the independent-samples t-test. Non-normally distributed continuous data were expressed as median (interquartile range) and were compared using the Mann-Whitney U test. Categorical data were presented as numbers (percentages) and were analyzed using the Pearson chi-square test. A significant overall Pearson chi-square test for the seasonal distribution of hospitalizations in RMPP patients was followed by post hoc pairwise comparisons with Bonferroni correction. To identify predictive factors for PB, significant continuous variables from the univariate analysis were first transformed into dichotomous variables based on the optimal cutoff points determined by the Youden index from the receiver operating characteristic (ROC) curves. These variables were then included in a multivariate logistic regression analysis to identify independent risk factors for PB in children with RMPP. The predictive performance of each independent risk factor was assessed by the area under the ROC curve. A *P*-value of less than 0.05 was considered statistically significant.

## Results

### Demographic and clinical information

**Fig. 3:**
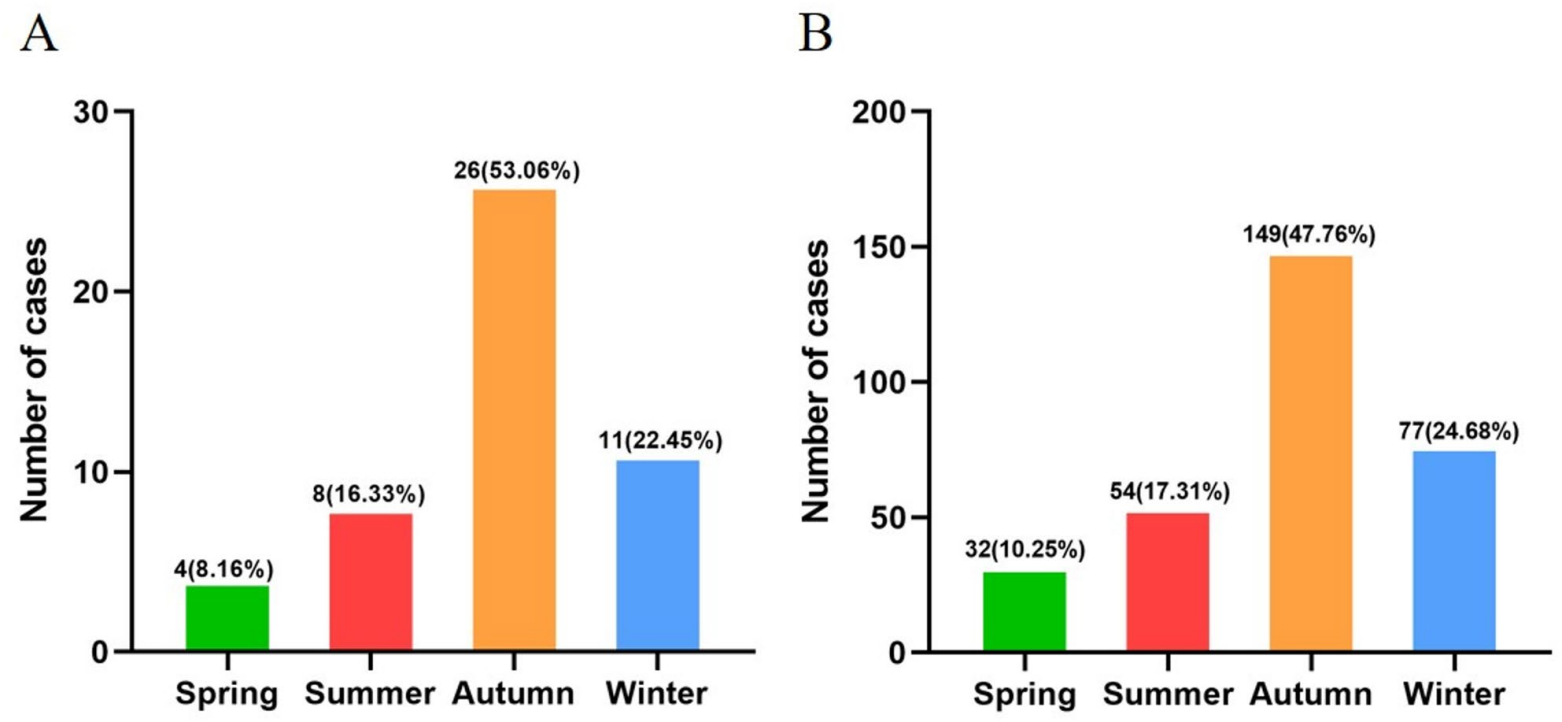
Seasonal distribution at onset in plastic bronchitis (PB) group (**A**) and non-PB group (**B**)

A total of 361 children with RMPP who underwent FB were enrolled in this study. Table 1 presented the demographic and clinical characteristics of the patients. The cohort comprised 49 cases (13.57%) in the PB group and 312 cases (86.43%) in the non-PB group. Male patients accounted for 53.06% (26/49) and 54.17% (169/312) of the PB and non-PB groups, respectively. The mean ages were comparable between groups (PB group: 7.30 ± 2.26 years; non-PB group: 7.02 ± 2.72 years). The Pearson chi-square tests revealed that the proportion of hospitalizations across the four seasons differed significantly between the PB and non-PB groups (all *P* < 0.001). Post hoc pairwise comparisons further demonstrated that, within both groups, the proportion of hospitalizations in autumn was significantly higher than that in any other season, with all *P*-values remaining below the adjusted significance level (Fig. 3). No significant intergroup differences were observed regarding gender distribution, age, and incident season (all *P* > 0.05). However, the incidence of extrapulmonary lesions was significantly higher in the PB group (*P* < 0.001). Among the 24 affected patients in this group, gastrointestinal involvement was most common (18 cases), followed by mucocutaneous (5), hematologic (4), circulatory (2), and muscular involvement (2). In the non-PB group, 58 patients developed extrapulmonary complications, primarily gastrointestinal (29 cases), mucocutaneous (9), and hematologic (8); circulatory, muscular, and renal manifestations each occurred in 4 cases. Significant differences were observed in treatment approaches between the two groups, with the PB group exhibiting significantly higher rates of multiple FB, alternative antimicrobial use, intravenous immunoglobulin (IVIG) administration, and glucocorticoid resistance (all *P* < 0.001). Figure 2C and D showed the chest CT features of a 7-year-old boy with RMPP complicated by PB before and after FB.

**Table 1:** Clinical features of children with RMPP between the PB group and the non-PB group

Characteristic	PB group (*n* = 49)	Non-PB group (*n* = 312)	Statistics(χ^2^/t)	*P* value
Male, *n*(%)	26 (53.06)	169 (54.17)	0.02	0.885
Age, years	7.30 ± 2.26	7.02 ± 2.72	0.60	0.548
Incident season, *n*(%)			0.54	0.910
Spring	4 (8.16)	32 (10.25)	-	-
Summer	8 (16.33)	54 (17.31)	-	-
Autumn	26 (53.06)	149 (47.76)	-	-
Winter	11 (22.45)	77 (24.68)	-	-
Extrapulmonary lesions, *n*(%)	24 (48.98)	58 (18.59)	22.28	<0.001
Multiple FB, *n*(%)	40 (81.63)	47 (15.06)	102.60	<0.001
Alternative antimicrobials, *n*(%)	23 (46.94)	33 (10.58)	42.72	<0.001
Corticosteroid resistance, *n*(%)	32 (65.31)	36 (11.54)	80.08	<0.001
IVIG, *n*(%)	14 (28.57)	18 (5.77)	24.51	<0.001

*MPP Mycoplasma pneumoniae* pneumonia, *PB *plastic bronchitis, *FB* fiberoptic bronchoscopy, *IVIG* intravenous immunoglobulin

### Analysis of the independent risk factors for PB occurrence in children with RMPP

**Fig. 4:**
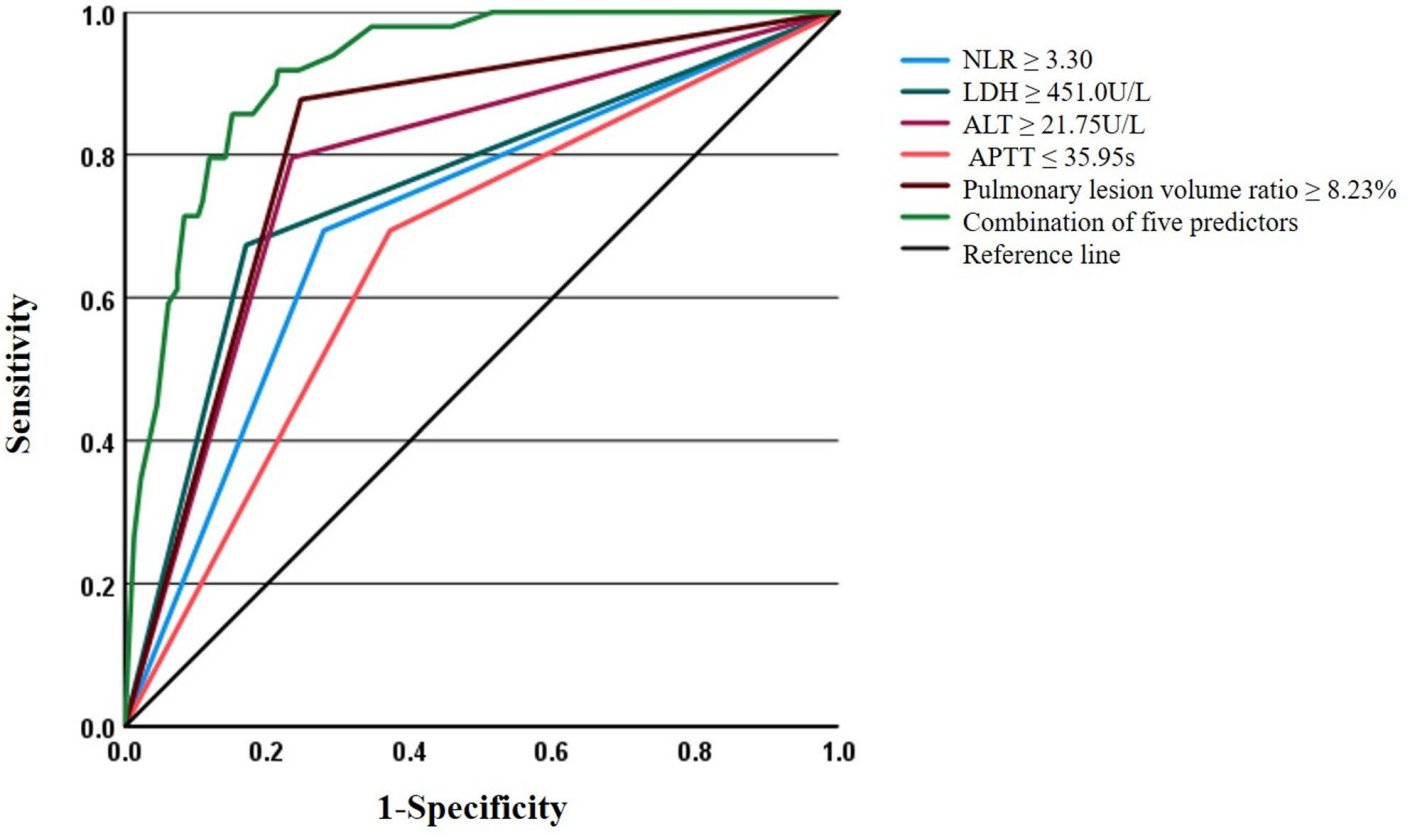
Receiver operating characteristic (ROC) curves of different predictors for plastic bronchitis (PB) in children with refractory *Mycoplasma pneumoniae* pneumonia (RMPP)

RMPP associated laboratory and chest CT parameters were compared by using univariate analysis, significant differences (*P*<0.05) were demonstrated in neutrophil-to-lymphocyte ratio (NLR), PLT, C-reactive protein (CRP), procalcitonin (PCT), erythrocyte sedimentation rate (ESR), LDH, aspartate aminotransferase (AST), alanine aminotransferase (ALT), prealbumin (PA), albumin (ALB), complement 3 (C3), complement 4 (C4), D-dimer (DD), activated partial thromboplastin time (APTT), proportion of neutrophils in BALF, pleural effusion, and volume ratio of pulmonary lesions between the PB and non-PB groups (Table 2). For continuous variables showing statistical significance, optimal cutoff values were determined through ROC curve analysis and Youden index calculation. These dichotomized variables were subsequently included in the multivariable logistic regression analysis: NLR ≥ 3.30, PLT ≤ 347.0 × 10^9^/L, CRP ≥ 11.34 mg/L, PCT ≥ 0.12ng/mL, ESR ≥ 44.50 mm/h, LDH ≥ 451.0U/L, AST ≥ 42.45U/L, ALT ≥ 21.75U/L, PA ≤ 103.50 mg/L, ALB ≤ 38.75 g/L, C3 ≤ 1.35 g/L, C4 ≤ 0.40 g/L, DD ≥ 895.0 µg/L, APTT ≤ 35.95s, proportion of neutrophils in BALF ≤ 35.50%, volume ratio of pulmonary lesions ≥ 8.23%. The final multivariate model identified five independent predictors of PB in pediatric RMPP cases (Table 3): elevated NLR (≥ 3.30 VS <3.30; OR, 2.92), increased LDH (≥ 451.0U/L VS <451.0U/L; OR, 4.91), higher ALT (≥ 21.75U/L VS <21.75U/L; OR, 5.68), greater pulmonary lesion volume ratio (≥ 8.23% VS <8.23%; OR, 8.83) and reduced APTT (≤ 35.95s VS >35.95s; OR, 4.20). Model performance was validated by a Hosmer-Lemeshow test (*P* = 0.93), indicating excellent calibration between predicted and observed outcomes. The predictive capability was further evaluated by ROC analysis (Fig. 4), with area under the curve (AUC) values of 0.71 for NLR ≥ 3.30, 0.75 for LDH ≥ 451.0U/L, 0.78 for ALT ≥ 21.75U/L, 0.66 for APTT ≤ 35.95s, and 0.82 for pulmonary lesion volume ratio ≥ 8.23%. The composite model combining all five predictors demonstrated superior diagnostic performance, with a sensitivity of 85.70%, specificity of 84.90%, and an AUC of 0.92 for predicting PB in children with RMPP.

**Table 2:** Laboratory values and chest CT features between the PB group and the non-PB group in children with RMPP

Variables	PB group	Non-PB group	Statistics(Z/χ^2^)	*P* value
WBC,×10^9^/L	7.91 (6.65–9.82)	7.66 (6.17–10.67)	0.41	0.681
NLR	3.97 (2.75–5.07)	2.36 (1.61–3.67)	5.18	<0.001
HB, g/L	120.0 (114.50–126.0)	122.50 (116.0–127.0.0.0)	1.50	0.133
EO, ×10^9^/L	0.06 (0.01–0.15)	0.05 (0.01–0.15)	0.62	0.536
PLT, ×10^9^/L	262.0 (217.0–327.50.0.50)	296.0 (242.0–371.75.0.75)	2.51	0.012
CRP, mg/L	20.68 (10.50–48.09.50.09)	10.37 (5.43–22.61)	4.35	<0.001
PCT, ng/mL	0.21 (0.12–0.47)	0.10 (0.06–0.16)	5.22	<0.001
ESR, mm/h	33.0 (21.0–49.50.0.50)	25.50 (15.25–38.0.25.0)	2.42	0.016
LDH, U/L	535.80 (364.0–703.20.0.20)	338.65 (273.40–424.63.40.63)	6.15	<0.001
AST, U/L	43.10 (32.15–76.45)	30.85 (25.33–39.18)	5.22	<0.001
ALT, U/L	28.40 (21.90–51.25.90.25)	14.70 (11.10–21.65.10.65)	6.19	<0.001
PA, mg/L	101.0 (82.50–127.50.50.50)	126.0 (101.0–169.0.0.0)	3.92	<0.001
ALB, g/L	38.70 (35.85–41.70)	41.80 (40.40–43.60)	5.33	<0.001
CK, U/L	76.40 (53.10–139.05.10.05)	78.20 (49.70–115.10.70.10)	0.28	0.779
CKMB, ng/ml	0.88 (0.59–1.22)	1.03 (0.76–1.35)	1.81	0.070
Troponin-t, pg/ml	3.10 (3.0–4.47.0.47)	3.15 (3.0–4.06.0.06)	0.53	0.600
C3, g/L	1.18 (1.12–1.31)	1.30 (1.12–1.43)	2.24	0.025
C4, g/L	0.33 (0.25–0.41)	0.38 (0.29–0.45)	2.25	0.025
DD, µg/L	1570.0 (955.0–4465.0.0.0)	690.0 (430.0–1050.0.0.0)	6.66	<0.001
APTT, s	34.20 (30.75–37.85)	37.30 (34.20–40.50)	4.35	<0.001
Fibrinogen, g/L	4.66 (4.19–5.27)	4.43 (3.88–5.09)	1.70	0.089
Cytological classification in BALF, %				
Neutrophil	57.0 (23.0–81.0)	70.0 (45.0–83.0)	2.24	0.025
Lymphocyte	3.0 (1.50–9.50)	2.0 (1.0–5.0)	1.62	0.105
Phagocyte	32.0 (13.0–68.50.0.50)	25.0 (15.0–50.75.0.75)	0.73	0.468
Chest CT features				
Consolidation, *n*(%)	45 (91.84)	298 (95.51)	0.56	0.456
Atelectasis, *n*(%)	13 (26.53)	53 (16.99)	2.58	0.108
Pleural effusion, *n*(%)	29 (59.18)	80 (25.64)	22.61	<0.001
Volume ratio of pulmonary lesions, %	18.86 (9.60–22.08.60.08)	6.75 (5.62–8.22)	9.32	0.000

*PB* plastic bronchitis, *RMPP* refractory *Mycoplasma pneumoniae* pneumonia, *WBC* white blood cell, *NLR* neutrophil-to-lymphocyte ratio, *HB* hemoglobin, *EO* eosinophil counts,* PLT* platelet, *CRP* C-reactive protein, *PCT* procalcitonin, *ESR* erythrocyte sedimentation rate, *LDH* lactic dehydrogenase, *AST* aspartate aminotransferase, *ALT* alanine aminotransferase *PA* pre-albumin, *ALB* albumin; *CK* creatine kinase, *CKMB* creatine kinase-myocardial band, *C3* complement3, *C4* complement4, *DD* D-dimer, *APTT* activated partial thromboplastin time, *BALF* bronchial alveolar lavage fluid

**Table 3:** Multivariate logistic regression analysis of risk factors for PB in children with RMPP

Independent variables	Logistic coefficient(β)	OR (95%CI)	*P* value
NLR ≥ 3.30	1.07	2.92 (1.13–7.53)	0.027
LDH ≥ 451.0U/L	1.59	4.91 (1.67–14.49)	0.004
ALT ≥ 21.75U/L	1.74	5.68 (2.08–15.51)	<0.001
APTT ≤ 35.95s	1.44	4.20 (1.63–10.82)	0.003
volume ratio of pulmonary lesions ≥ 8.23%	2.18	8.83 (1.68–46.33)	0.010

*PB* plastic bronchitis, *RMPP* refractory *Mycoplasma pneumoniae* pneumonia, OR odds ratio, *NLR* neutrophil-to-lymphocyte ratio,* LDH* lactic dehydrogenase, *ALT* alanine aminotransferase, *APTT* activated partial thromboplastin time

### Percentage of PB patients in the RMPP scoring groups

**Fig. 5:**
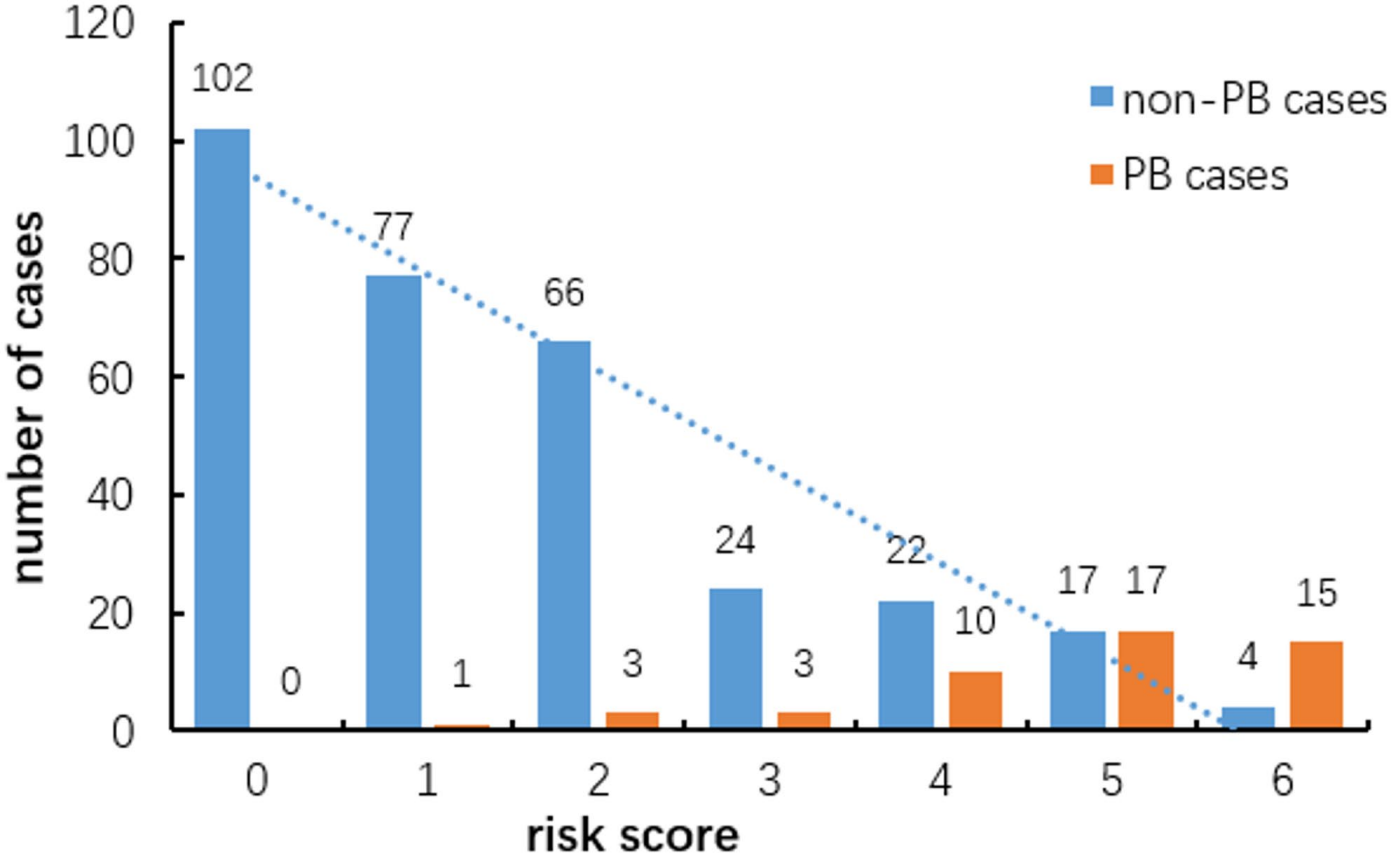
Correspondence between the scoring model and the occurrence of plastic bronchitis (PB)

The five independent risk predictors were scored based on odds ratio (OR) values, with pulmonary lesion volume ratio ≥ 8.23% scoring 2 points, NLR ≥ 3.30, LDH ≥ 451.0U/L, ALT ≥ 21.75U/L, and APTT ≤ 35.95s all scoring 1 point. Using this scoring system, we stratified RMPP patients into three risk categories: high-risk group (5–6 points), middle-risk group (3-4points), and low-risk group (0-2points). Among them, 53 cases were in the high-risk group and 32 cases (60.38%) were caused by PB. There were 59 cases in the middle-risk group, 13 cases (22.02%) with PB, 249 cases in the low- risk group, and 4 cases (1.61%) with PB (Fig. 5).

## Discussion

In recent years, there has been growing recognition and reporting RMPP in children, with bronchoscopy revealing an increasing number of cases complicated by PB [12]. Once PB is suspected, prompt removal of bronchial casts through FB is essential to relieve airway obstruction and prevent the progression of sequelae. This study identified several independent risk factors for PB in RMPP, including a higher pulmonary lesion volume ratio (≥ 8.23%), reduced APTT (≤ 35.95s), elevated NLR (≥ 3.30), and increased levels of LDH (≥ 451.0U/L) and ALT (≥ 21.75U/L).

While prior studies have linked pleural effusion and atelectasis to PB [9, 12], and our previous study demonstrated that the pulmonary lesion volume ratio can be used to assess the risk of PB in children with MPP [19]. However, its utility has not been further discussed in RMPP patients. Chest CT is an effective tool for assessing pulmonary involvement. In this study, we analyzed CT images and quantified pulmonary lesion volume using 3D Slicer software. Our findings indicate that RMPP patients with a higher pulmonary lesion volume ratio are at greater risk of PB occurrence. Notably, the incidence of lung consolidation in RMPP cases is significantly higher than in general cases, reflecting more severe pulmonary involvement [4]. When PB develops, airway obstruction can lead to extensive atelectasis in the affected lung segments. Thus, accurate measurement of pulmonary lesion volume may improve risk stratification for PB in RMPP patients.

LDH is a cytoplasmic enzyme released into the serum following cell lysis or membrane damage [20]. In cases of RMPP-associated PB, elevated LDH may result from pulmonary or cardiac tissue injury secondary to airway inflammation, airflow obstruction, increased respiratory effort, hypoxemia, or myocardial involvement. Consistent with other studies [9, 12], we found significantly higher serum LDH levels in RMPP children with PB compared to those without PB. Choi et al. also observed marked increases in both LDH and ALT levels in RMPP patients [21]. Notably, elevated ALT during MP infection underscores the significance of extrapulmonary injury markers in forecasting poor outcomes in MPP [22]. Consistent with these observations, our study identified ALT as an independent risk factor for PB in RMPP. Furthermore, the PB group exhibited a higher incidence of extrapulmonary complications than the non-PB group, reinforcing the association between systemic injury and PB development. MP infection can induce both excessive immune responses and activation of coagulation pathways through multiple mechanisms, resulting in coagulation abnormalities, thrombosis, and subsequent organ damage. In MPP patients, these coagulation disturbances typically present as elevated fibrinogen and DD levels along with shortened APTT [23–25]. Liver dysfunction may reduce antithrombin activity, contributing to hypercoagulability [26, 27], which aligns with our findings of concurrent liver injury and shortened APTT in PB patients. Supporting these observations, Wang et al. [25] reported negative correlations between ALT and APTT, positive correlations between ALT and DD in MPP children, and significantly elevated fibrinogen and DD levels in MPP children with lung consolidation.

Research has shown that neutrophilia significantly increases the risk of PB [9, 28], while lymphopenia exacerbates airway injury in RMPP [13]. NLR, the ratio of neutrophil to lymphocyte counts in peripheral blood, reflecting innate-adaptive immune balance, emerged in our study as an independent PB risk factor, corroborating Mu et al.‘s findings [29]. The underlying mechanism may involve an exaggerated pulmonary inflammatory response in pediatric patients with PB, characterized by excessive pro-inflammatory cytokines that promote neutrophil release from bone marrow. These neutrophils then exacerbate airway inflammation and mucus plug formation through multiple pathways, including the release of reactive oxygen species, proteolytic enzymes, and neutrophil extracellular traps [28]. Concurrently, MP may suppress lymphocytes through proliferation inhibition or apoptosis induction. These comprehensive factors lead to an increase in NLR, indicating immune dysregulation and disease severity, which can aid in early PB identification and guide immune regulation or anti-inflammatory treatment. Refractory cases often require early glucocorticoid administration following antibiotic therapy, typically at higher doses than standard regimens [30]. Consistent with this, our PB group exhibited higher resistance to conventional doses of glucocorticoids, with consequently increased use of IVIG.

Our study demonstrated that combining the pulmonary lesion volume ratio with key laboratory markers significantly improves PB detection in RMPP patients, achieving 85.70% sensitivity and 84.90% specificity. We developed a scoring system based on these five independent predictors, weighted by their OR values: pulmonary lesion volume ratio ≥ 8.23% (2 points), NLR ≥ 3.30 (1 point), LDH ≥ 451.0 U/L (1 point), ALT ≥ 21.75 U/L (1 point), and APTT ≤ 35.95 s (1 point). Patients scoring 5–6 points showed strong PB likelihood, with the maximum score indicating highest risk.

This study has several limitations that should be considered. First, its retrospective design at a single center with a relatively small sample size may introduce selection bias. This is particularly relevant as children with RMPP who did not undergo bronchoscopy were excluded, potentially limiting the generalizability of our results to the entire RMPP population. Future prospective, multi-center studies with larger, consecutively enrolled cohorts are necessary to mitigate this bias and validate our findings. Second, the timing of laboratory sample collection was not standardized and varied following disease onset. This heterogeneity could lead to measurement time bias, as biomarker levels may fluctuate at different stages of the disease. This is an inherent challenge in retrospective studies. Therefore, we should clearly record the time from onset to sampling of all included patients and consider this factor in the multivariate regression model to minimize such bias. Despite these limitations, the clear associations and the proposed scoring system provide a valuable foundation for further investigation.

## Conclusion

In summary, this study identified a suite of objective and readily available parameters—NLR, LDH, ALT, APTT, and pulmonary lesion volume ratio—as independent predictors for PB in pediatric RMPP. The integration of these markers into a practical scoring system provides clinicians with a powerful tool for early risk stratification, enabling timely bronchoscopic intervention and optimized management for high-risk patients. To confirm its generalizability and clinical impact, the prospective, multicenter validation of this scoring system is a necessary and critical next step.

## Data Availability

All analyzed datasets are available from the corresponding author upon request.

## Abbreviations

PB: Plastic Bronchitis
MPP: *Mycoplasma Pneumoniae* Pneumonia
MP: *Mycoplasma Pneumonia*
RMPP: Refractory *Mycoplasma Pneumoniae* Pneumonia
FB: Fiberoptic Bronchoscopy
WBC: White Blood Cell
NLR: Neutrophil-to-Lymphocyte Ratio
HB: Hemoglobin
EO: Eosinophil Counts
CRP: C-Reactive Protein
PCT: Procalcitonin
PLT: Platelet
ESR: Erythrocyte Sedimentation Rate
PA: Pre-Albumin
ALB: Albumin
CK: Creatine Kinase
CKMB: Creatine Kinase-Myocardial Band
C3: Complement3
C4: Complement4
DD: D-dimer
APTT: Activated Partial Thromboplastin Time
LDH: Lactate Dehydrogenase
AST: Aspartate Transaminase
ALT: Alanine Transaminase
IVIG: Intravenous Immunoglobulin
AUC: Area Under Curve
ROC: Receiver Operating Characteristic
BALF: Bronchial Alveolar Lavage Fluid
OR: Odds Ratio
IL-6: Interleukin-6
